# Shedding Light on the Venom Proteomes of the Allergy-Relevant Hymenoptera *Polistes dominula* (European Paper Wasp) and *Vespula* spp. (Yellow Jacket)

**DOI:** 10.3390/toxins12050323

**Published:** 2020-05-14

**Authors:** Johannes Grosch, Christiane Hilger, Maria Beatrice Bilò, Stephanie Kler, Maximilian Schiener, Gunnar Dittmar, François Bernardin, Antoine Lesur, Markus Ollert, Carsten B. Schmidt-Weber, Simon Blank

**Affiliations:** 1 Center of Allergy and Environment (ZAUM), Technical University of Munich, School of Medicine and Helmholtz Center Munich, German Research Center for Environmental Health, 85764 Munich, Germany; johannes.grosch@helmholtz-muenchen.de (J.G.); schiener.maximilian@gmail.com (M.S.); csweber@tum.de (C.B.S.-W.); 2 Department of Infection and Immunity, Luxembourg Institute of Health (LIH), 4354 Esch-Sur-Alzette, Luxembourg; christiane.hilger@lih.lu (C.H.); Stephanie.Kler@lih.lu (S.K.); markus.ollert@lih.lu (M.O.); 3 Department of Clinical and Molecular Sciences, Polytechnic University of Marche, Ancona and Allergy Unit, Department of Internal Medicine, University Hospital of Ancona, 60126 Ancona, Italy; m.b.bilo@staff.univpm.it; 4 Quantitative Biology Unit, Luxembourg Institute of Health (LIH), 1445 Strassen, Luxembourg; gunnar.dittmar@lih.lu (G.D.); Francois.Bernardin@lih.lu (F.B.); antoine.lesur@lih.lu (A.L.); 5 Department of Dermatology and Allergy Center, Odense Research Center for Anaphylaxis, University of Southern Denmark, 5000 Odense, Denmark

**Keywords:** allergen, Hymenoptera venom allergy, European paper wasp, *Polistes dominula* venom, venom proteome, *Vespula* spp. venom, yellow jacket

## Abstract

Allergic reactions to stings of Hymenoptera species can have serious or even fatal consequences. If the identification of the culprit insect is possible, venom-specific immunotherapy effectively cures Hymenoptera venom allergies. Although component-resolved diagnostics has strongly evolved in recent years, the differentiation between allergies to closely related species such as *Polistes dominula* and *Vespula* spp. is still challenging. In order to generate the basis for new diagnostic and therapeutic strategies, this study aims at resolving the venom proteomes (venomes) of these species. The venoms of *P. dominula* and *Vespula* spp. (*V. germanica*, *V. vulgaris*) were analyzed by liquid chromatography-mass spectrometry. Resulting proteins were characterized regarding their function, localization and biochemical properties. The analyses yielded 157 proteins in *Vespula* spp. and 100 in *P. dominula* venom; 48 proteins, including annotated allergens, were found in both samples. In addition to a variety of venom trace molecules, new allergen candidates such as icarapin-like protein and phospholipase A2 were identified. This study elucidates the venomes of closely related allergy-eliciting Hymenoptera species. The data indicates that relying on marker allergens to differentiate between *P. dominula* and *Vespula* spp. venom allergy is probably insufficient and that strategies using cross-reactive major allergens could be more promising.

## 1. Introduction

Allergies directed against Hymenoptera venoms are the most frequent cause of systemic IgE-mediated hypersensitivity reactions—also known as anaphylaxis—in adults [1]. Venom-specific immunotherapy (VIT) is currently the only known curative therapy, but its efficacy greatly depends on the correct identification of the culprit insect. However, in some patients a proper diagnosis is not possible despite thorough anamnesis, skin allergy tests, specific IgE (sIgE) measurement to whole venom extracts and cellular tests such as, for example, the basophil activation test (BAT). One problem-solving approach is to improve component-resolved diagnostics (CRD) by adding novel allergens to the diagnostic palette [2,3]. Various studies on insect venoms with a main focus on the order Hymenoptera yielded a variety of new allergens [4]. Nevertheless, discriminating venom allergy against closely related species, as for example European paper wasp (*Polistes dominula)* from the yellow jacket (*Vespula* spp.) venom allergy, remains difficult.

*P. dominula*, a social wasp originating from Southern Europe, North Africa and temperate parts of Asia, was introduced to the United States in 1970. Over a period of 20 years, *P. dominula* spread from the northeast to the west coast [5]. *P. dominula* was further observed as far north as the Netherlands in the 1980s [6] and in 2008 in South Africa [7]. Sightings in Australia [8] and South America [9] completed the spread of *P. dominula* on all continents except the Antarctic. This invasion is likely to contribute to an increase in allergies to *P. dominula* venom (PDV), as previously described for, e.g., *V. vulgaris* in Alaska [10,11].

*V. vulgaris* as well as *P. dominula* belong to the Vespidae, one of the three allergy-relevant families in the order Hymenoptera besides the Apidae and the Formicidae. While prominent members of the Apidae—the honeybees (*Apis mellifera*)—are known to lose their stinger, the venom gland and subsequently their lives after stinging, wasps, hornets and ants can potentially inflict more than one sting without dying [12]. With each sting, a species-dependent amount of venom is injected into the wound; the protein content per sting varies between 1.7 to 3.1 µg in yellow jackets, around 17 µg in *Polistes* spp. and up to 59 µg in honeybees [13,14]. In addition, the amount of injected venom can be manipulated by the insect itself.

Hymenoptera venoms comprise a variety of different bioactive compounds from substance classes as low molecular weight molecules, peptides and proteins. Small molecules like histamine, serotonin or dopamine, for instance, can be found in all three allergy-relevant Hymenoptera families. However, the vast majority of compounds present in insect venoms are proteins and peptides. In honeybee venom (HBV), for example, the 26 amino acid-long polypeptide melittin (Api m 4) accounts for more than 50% of total dry venom. Numerous other venom-derived peptides such as, for example, mastoparan from *V. vulgaris* or Polybia-MP1 from *Polybia paulista* are part of ongoing research as substitution for antibiotics [15] and in cancer treatment [16].

The main triggers of allergies to Hymenoptera venoms are proteins. The best-characterized insect venom in allergy, HBV, is thought to have 113 protein components [17], while so far only 46 were found in *Solenopsis invicta* [18], a fire ant species and, therefore, part of the Formicidae family. However, some of these proteins have no apparent function in the venoms and are occasionally referred to as “venom trace molecules” [17]. A recent study on the venom proteome of *P. paulista* reports the presence of 1673 proteins, of which 1049 were uncharacterized, 329 involved in housekeeping, and 295, about 18%, asserted venom proteins with proposed venom function [19]. This remarkable discrepancy is most likely not solely due to species-dependent differences or improved methodological sensitivity but rather an artifact originating from the usage of different databases comprising the same or highly similar proteins from different species. Therefore, during peptide alignment, several proteins for the same input peptide are reported.

Understanding the composition of insect venoms is the first step towards identification of novel allergens. Hymenoptera venom-allergic patients show sensitization against a variety of proteins from different families. Prominent representatives are, for example, the phospholipases A1 (e.g., Pol d 1, Ves v 1) and A2 (e.g., Api m 1), which are regarded as myo- or neurotoxins [20]. Hyaluronidases (e.g., Api m 2, Ves v 2) cleave hyaluronan in the extracellular matrix on the site of stinging and, therefore, serve to distribute the venom in the victim [21,22]. Antigen 5 proteins, which are part of, for instance, *V. vulgaris*, *P. dominula* and *P. paulista* venoms, have been reported to be highly cross-reactive, although their function remains unknown [23]. As was described earlier, a plethora of other proteins was reported in literature as part of Hymenoptera venoms and some have since been characterized as allergens [4]. 

In this study we analyzed the venom proteomes (venomes) of *Polistes dominula* and *Vespula* spp. (*V. vulgaris* and *V. germanica*) using different proteomic approaches and characterized identified proteins in silico regarding their function, localization and biochemical properties. These species are of major importance as elicitors of venom allergy and, hence, detailed knowledge of the venom proteomes might contribute to the development of advanced diagnostic and therapeutic strategies.

## 2. Results

**Figure 1:**
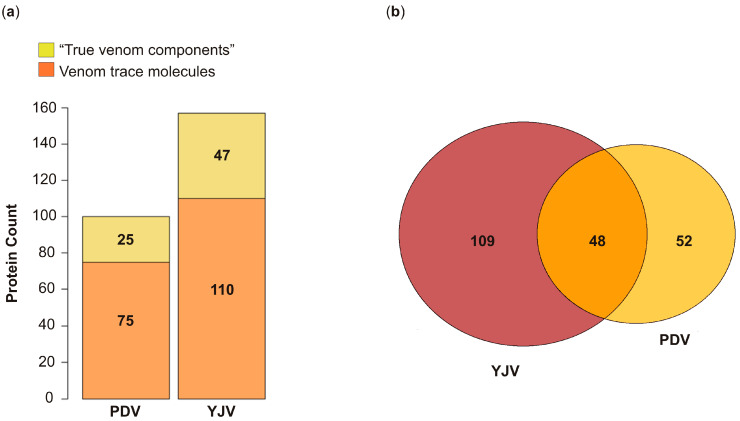
Identified proteins in *Polistes dominula* and *Vespula* spp. venom. (**a**) Total number of proteins identified in the venoms. The proteins are divided on the one hand into venom components exhibiting a signal peptide for transport in the extracellular matrix, are annotated as allergens or have a known function in the venom (“true venom components”) and on the other hand into venom trace molecules lacking a signal peptide. (**b**) Number of total proteins found in YJV and PDV and the overlap region containing proteins identified in both venoms. PDV, *Polistes dominula* venom; YJV, yellow jacket venom.

The analysis of mass-spectrometrically identified peptides of *Vespula* spp. venom (a mixture of the venoms of the closely related species *V*. *vulgaris* and *V*. *germanica* venom) yielded 1140 proteins. After removing double entries caused by similar proteins from different Hymenoptera species, 157 proteins were left, while *P. dominula* venom returned 100 proteins (Figure 1a). An overlap of 48 yields 109, respectively 52 unique venom proteins (Figure 1b). The two datasets were further divided into three distinct groups: already annotated allergens, secreted proteins/proteins with known function in the venom (these two groups are classified as “true venom components”) and venom trace molecules without signal peptide. 

**Figure 2:**
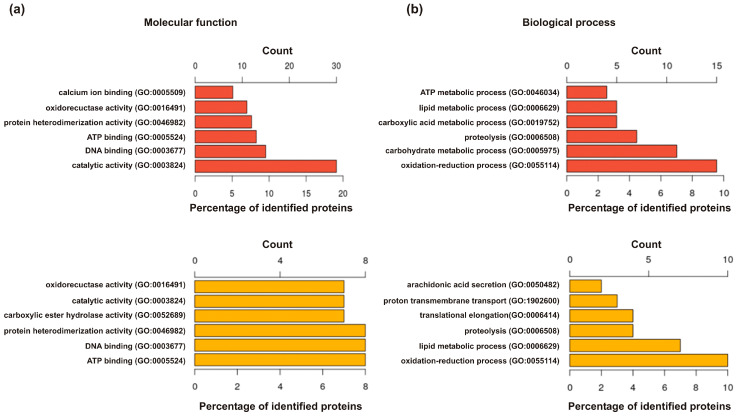
The most frequently found Gene Ontology (GO) terms for (**a**) molecular function and (**b**) biological process of proteins from yellow jacket venom (red) or *Polistes dominula* venom (yellow). The absolute count is given on the *x*-axis above the plots, the relative number in [%] on the lower axis.

Figure 2 shows the most common Gene Ontology (GO) terms for proteins identified in yellow jacket venom (YJV) and/or *Polistes dominula* venom (PDV). This allows a quick comparison of protein functions at the molecular level (Figure 2a) and an assessment of the biological processes (Figure 2b) in which they are involved. While the absolute numbers differ due to the varying amount of proteins identified in the two venoms, the relative abundance is quite comparable. However, YJV seems to consist of up to 20% of proteins with catalytic activity which is, compared to PDVs 7%, quite remarkable. This discrepancy is probably due to greater contamination with housekeeping proteins in the YJV preparation, since, with the exception of the venom hyaluronidases, metabolically active enzymes are mainly given this GO term. Overall, there only seem to be minor differences in the composition of the two venoms in terms of molecular functions or biological processes of the proteins.

### 2.1. Identification of Already Annotated Allergens

The analyses returned all already annotated allergens from the venoms investigated here (Table 1) [24] apart from the high molecular weight allergen vitellogenin (Ves v 6). Except for the PDV serine protease Pol d 4, all of them are part of the aforementioned overlap region (Figure 1b).

The most prominent *Vespoidea* allergens are antigens 5 which belong to the CAP (cysteine-rich secretory proteins, antigen 5, and pathogenesis-related 1 proteins) superfamily. While most representatives of the CAP superfamily are secreted and function as endocrine or paracrine modulators [25], the role of Hymenoptera venom antigen 5 remains elusive. Various functions in animal venoms or saliva have been proposed, such as altering platelet aggregation and thus modulation of the immune system [26] or the blocking of ion channels, including ryanodine receptors, Ca^2+^ and K^+^ channels [27,28,29]. Nevertheless, their role in the envenoming process after stings of Hymenoptera continues to be unclear.

Other relevant allergens in various *Vespoidea* venoms are phospholipases A1 (PLA1), which hydrolyze the sn-1 position of phospholipids. Since phospholipids are part of pro- and eukaryotic cell membranes, venom phospholipases are known to disrupt cells. PLA1 of PDV (Pol d 1) and yellow jacket venom (YJV) (Ves v 1) represent important major allergens of both venoms [30].

Dipeptidyl peptidases IV (DPP IV) are aminopeptidases that cleave (pro)peptides at the N-terminus of proteins and separate dipeptides from the main chain, thereby, activating or inactivating the substrate [31]. For instance, in HBV DPP IV (Api m 5) [32] catalyzes the reaction from promelittin to melittin (Api m 4), a cytotoxic polypeptide [33]. A similar mode of action is known for DPP IV from *V. vulgaris* (Ves v 3) and the venom-polypeptide mastoparan [34]. With the activity-triggering enzyme being part of the venom, the host insect probably protects itself against unintended and undirected toxic effects of the peptide substrates. Although DPP IV was recently characterized as a relevant allergen (Pol d 3) in PDV [35], to our knowledge, there is no propeptide identified, which shows the required prerequisites to be a susceptible substrate. Hence, its function in PDV so far remains elusive.

Hyaluronidases cleave hyaluronan, the most abundant glycosaminoglycan in vertebrates’ extracellular matrix and thereby promote the spread of the venom at the site of puncture [21]. Hyaluronidases are common components of Hymenoptera venoms and have been annotated as allergens for eight species [24], amongst others for *A. mellifera*, *P. paulista* and *V. vulgaris*. Sequence identity of the newly identified *P. dominula* hyaluronidase Pol d 2 and venom hyaluronidases of aforementioned species lies around 53% (Api m 2, query cover 90%), 91% (Poly p 2, query cover 79%), 74% (Ves v 2.0101, query cover: 90%) and 57% (Ves v 2.0201, query cover: 92%).

The trypsin-like serine protease from PDV Pol d 4 was first described, and its allergenic properties assessed, in 2003 [36,37]. Its role in PDV is probably linked to its ability to initiate coagulation and damage tissue, which is, for instance, known from serine proteases present in different snake venoms [38]. No homologous protein was identified in YJV.

### 2.2. Identification of Secreted Proteins and Proteins with Known Function in the Venom

Using an in silico prediction method [39], the primary sequences of all proteins were examined for the presence of signal peptides for the extracellular space. Since all genuine venom components need to be shuttled out of the cell and, therefore, must have a signal peptide, the proteins listed in Table 2 were considered putative venom proteins.

Interestingly, an icarapin-like protein was identified exclusively in PDV. Icarapin (Api m 10) is a major allergen from HBV with unknown molecular function [40,41]. Icarapin was described as a marker allergen for HBV allergy [3,42], despite its unstable nature. Its name is an artificial term combining “Icarus” and the genus name “Apis” and indicates its rapid degradation. The here reported icarapin-like protein of PDV shows sequence identity of 43% to the canonical sequence of Api m 10. So far, no studies have been carried out to assess the allergenicity of icarapin-like protein from PDV.

Phospholipases A2 (PLA2) have already been described in various Hymenoptera (*Apis* spp., *Bombus terrestris*) but also in snake and spider venoms [20,43] and act as neuro- and myotoxins by hydrolyzing membrane phospholipids of motor nerves and subsequently leading to cell lysis [20]. Here, two different isoforms with a sequence identity of 99% are reported in PDV and one of these isoforms in YJV. The two PDV PLA2 isoforms differ in one amino acid position and show about 45% sequence identity to Api m 1, the PLA2 and major allergen from HBV [44].

Three different variants of protein lethal (2) essential for life-like (PLELL) were found in PDV. The proteins vary in length (246, 201 and 196 amino acids) and in the presence of signal peptides (two without a signal peptide and, therefore, listed in Table S2). With the identity of 41% and 39%—using the 246 amino acid variant as a query—the proteins only show little similarity at the level of primary sequences. This might be due to gene duplication by unequal crossing over, since the different proteins are encoded on adjacent genes. PLELLs are part of the Hsp20 family, a cluster of small heat-shock proteins (sHSP) involved in chaperoning. What specific role PLELL in Hymenoptera venom plays is unknown.

Vascular endothelial growth factor C from PDV belongs to the PDGF family (PF00341). Representatives of the PDGF family are a further part of other venoms such as VEGF Fs in snake [45] or PVF 1 in HBV [46]. The latter has been subject to allergy research, as it has been demonstrated, that about 40% of HBV-allergic patients have sIgE directed against this venom component but no downstream FcεRI crosslinking occurs [47]. In addition to their role as allergens that trigger Th2 immune responses, it should always be borne in mind that the majority of true venom components are also toxic substances that can directly modulate physiological processes in the host.

Most of the proteins exclusively identified in YJV (Table 2) are also predicted for *Polistes dominula* and, therefore, unlikely to be specific for *Vespula* spp. Moreover, the descriptions of many of these proteins imply that they have no venom-specific function but have to be considered venom trace molecules that are secreted into the extracellular matrix of the venom gland [17].

In addition, some small peptides were identified in the two venoms, namely mastoparan, vespakinin and vespulakinin in YJV and dominulin A and B in PDV. Although the prediction did not show any leading sequences, these peptides are likely to be “true venom components” and, therefore, listed in Table 2. As not all underlying shuttle mechanisms are fully elucidated and precursor proteins are sometimes unknown, the discrimination between actively transported venom components and household contaminants is not always straightforward. Mastoparans are closely linked to DPP IV (Ves v 3) as their progenitors contain N-terminal sequences that are released after cleavage of the signal peptide and are DPP IV substrates. The shuttling mechanisms are, therefore, based on transit peptides present in progenitor proteins, which explains how mastoparans end up in the venom despite the lack of a signal peptide [34]. Nevertheless, no precursor was found in the proteome analysis presented here, a fact that is probably due to the missing genomic data of *Vespula* spp. Dominulins A and B from PDV are small peptides of 17 amino acids with 12 residues being identical. These peptides show antibacterial properties and are probably closely related to mastoparans [48]. Drawing a connection between dominulins and *P. dominula* DPP IV (Pol d 3) seems obvious, but so far no link has been reported. In addition, no precursor of dominulin A or B is known or predicted. In preceding studies, the two variants were found both on the cuticle and in the venom, although the occurrence in the latter was irregular [48]. While the presence of mastoparan in the venom and its role as a true venom component appears to be clear [49], dominulins remain elusive. Vespulakinins/vespakinins are vasoactive glycopeptides containing the nine amino acid long bradykinin [50]. Upon injection in the victim, this peptide has mainly three different pharmacological effects: the release of histamine from mast cells, lowering blood pressure [50] and the induction of pain [51]. The here presented analysis yielded vespulakinin/vespakinin in YJV, while polisteskinin, a rather similar peptide described in the venoms of *Polistes* spp., was not detected in PDV. However, the detection of such short peptides is rather improbable in a shotgun-run. Most proteins are identified with several peptides. A clear confirmation would require a targeted proteomics experiment.

Overall, 47 and 25 proteins that are secreted or have a known function in the venom were identified in YJV and PDV, respectively, with an overlap of 14 proteins. These include the proteins listed in Table 2 as well as the annotated allergens from Table 1.

### 2.3. Identification of Venom Trace Molecules without Signal Peptides

The presence of proteins without signal peptides in Hymenoptera venoms was reported numerous times [17,18,19]. These proteins are described as venom trace molecules that are unintentionally secreted by the venom gland cells due to insufficient protein recycling, cell damage or co-secretion with other compounds [17]. In YJV and PDV, 110 and 75 of these proteins were identified, respectively (Figure 1a). These venom trace molecules comprise a variety of proteins such as heat shock proteins (HSP) or members of different enzyme classes (hydrolases, isomerases, lyases, oxidoreductases, transferases and translocases) (Tables S1 and S2). Since these proteins may have functions in cells of the venom gland as well as in the venom, identifying venom contaminants released from surrounding tissue is difficult (Table S3 lists the GO annotations for all identified proteins of PDV and YJV).

## 3. Discussion

In recent years, growing knowledge about the venom composition of different allergy-relevant Hymenoptera species by proteomic approaches has increasingly shed light on the identity of relevant venom allergens of the different species [4]. Moreover, the identification of particular important venom allergens has paved the way from a venom extract-based allergy diagnostics to the use of individual allergens and opened the era of component-resolved diagnostics [52]. Nowadays, CRD has already proven to allow a more reliable diagnostics of Hymenoptera venom-allergic patients including a straightforward discrimination between HBV and vespid venom allergy [2,3]. Classic CRD requires the use of species-specific marker allergens in order to be able to distinguish allergies to venoms from closely related Hymenoptera species. To date, HBV is clearly the best characterized Hymenoptera venom, whereas detailed proteomic data of PDV and YJV were not available so far. Hence, this study analyzed the venomes of these species by mass spectrometric analyses.

Due to the lack of genomic data of *Vespula* spp., a Hymenoptera database combined with a database comprising predicted and identified *Polistes dominula* and *Vespula* spp. proteins were used for the identification of *Vespula* spp. venom proteins. Although the databases used comprise 601.844 entries from various species, this search might miss true species-specific proteins. Nevertheless, 109 unique proteins were identified in YJV (from 157 proteins in total) that were not found in PDV, out of which 33 show a predicted signal peptide. An additional 14 secreted proteins, including most of the relevant annotated allergens, have highly homologous variants in PDV and are part of the venome overlap. The analysis of PDV yielded 52 out of 100 proteins not found in YJV, out of which 11 have a predicted signal peptide. The higher number of proteins identified in YJV might be due to a lower purity of the venom preparation, as most of these proteins seem to be household contaminants from the surrounding tissue. Of note, proteins identified by only one peptide (marked in Table 2 and Tables S1 and S2) have an increased likelihood of being false positives, although the peptides passed the score thresholds. However, most of these proteins represent household contaminants or venom trace molecules with potentially negligible allergological relevance.

So far, species-specific marker allergens that allow an unequivocal discrimination between PDV and YJV allergy are missing. Especially in Southern Europe where double sensitization to YJV and PDV is more frequent than that to YJV/PDV and HBV [53,54,55], the discrimination between PDV and YJV allergy is still challenging. This is mostly due to the high degree of cross-reactivity between the identified major allergens of the two venoms. For instance, antigens 5, which are described as major allergens in at least 19 different stinging insects [24], show pronounced cross-reactivity and, hence, cannot be used as reliable marker allergens in CRD [23]. The same holds true for other relevant allergens such as PLA1 or DPP IV [30,32,35]. As *P. dominula* hyaluronidase was identified for the first time, its relevance as an allergen so far remains elusive. No sensitization patterns of Pol d 4 (a homologous protein was not identified in YJV) have been published so far. However, unpublished data hints to a role of Pol d 4 as minor allergen with sensitizing potential only in a minority of PDV-allergic patients and, therefore, with restricted relevance for CRD.

In our opinion, some of the here reported secreted proteins, such as PLA2 or icarapin-like protein, are promising new candidates to be tested for their allergenicity as their homologues are known as major allergens in other Hymenoptera venoms such as HBV [44]. Although these potential allergens are not likely to act as marker allergens for primary sensitization, the availability of additional cross-reactive allergen pairs and comparative sIgE measurements may add value for the discrimination of allergies to the different species.

According to their description, the majority of the remaining newly identified venom proteins that contain signal peptides for extracellular transport are likely to be venom trace molecules with no venom-specific function that are released from the extracellular space of the venom gland [17]. It can only be speculated if these venom trace molecules have relevant allergenic potential as their presence and amount in the venom may show huge variability. The same holds true for venom trace molecules lacking signal peptides for active extracellular transport that are accidentally released in the venom sac.

Due to the fact that all of the so far identified major allergens of YJV and PDV exhibit pronounced cross-reactivity and that new candidates, which could act as species-specific marker allergens, seem to be rare, the likelihood of developing CRD approaches comparable to those for the discrimination of HBV and YJV allergy for reliable differentiation between YJV and PDV allergy is very low.

However, a recent study reported that the measurement of the relative levels of sIgE to the major allergens phospholipases A1 (Ves v 1 and Pol d 1) and antigens 5 (Ves v 5 and Pol d 5) of YJV and PDV is able to detect the most probable sensitizing species in 69% of allergic patients with double sensitization [30]. A subsequent study showed that the detection of sIgE against the same four allergens could determine the correct venom for immunotherapy in the majority, but not in all patients [56]. Unfortunately, not all required allergens are available for the most commonly used sIgE assay platforms. Limitations of the currently commercially available components for CRD to distinguish between YJV and PDV allergy in double-positive patients was demonstrated by the fact that a good accordance between antigen 5-based CRD and CAP-inhibition assays can only be achieved when the value of sIgE in kUA/L to Ves v 5 was about twice those to Pol d 5 and vice versa [57,58]. However, a later multicenter study did not find any agreement between CAP-inhibition test results and double sIgE values of Ves v 5 over Pol d 5 or vice versa [59]. Hence, the availability of additional cross-reactive major allergens of YJV and PDV for routine diagnostics such as PLA1 and DPP IV surely would add value for uncovering primary sensitization in vespid venom allergic patients and to distinguish true mono- from true double-sensitization. Moreover, the use of venom extracts or single allergens in basophil activation tests and the comparison of dose-response curves are likely to contribute to the identification of primary sensitization in Hymenoptera venom allergy [23,60,61].

## 4. Materials and Methods

### 4.1. Sample Preparation

Capillary-extracted venoms of *Polistes dominula* and *Vespula* spp. (*V. vulgaris* and *V. germanica*) (Entomon, Florence, Italy) were purified and trypsin-digested using both the SP3 on-bead and the polyacrylamide in-gel method.

The SP3 method was performed according to Hughes et al. protocol [62]. Briefly, the venom extracts were clarified by centrifugation and the cysteine disulphide bonds were reduced and alkylated with dithiothreitol and iodoacetamide, respectively. SP3 beads (Thermo Fisher Scientific, Waltham, Massachusetts, USA) were added to the samples to capture the proteins and washed three times with 70% ethanol solution. Proteolysis was performed by incubating the beads with a trypsin/lysC mixture (Promega, Madison, WI, USA) overnight at 37 °C. After digestion, peptides were captured on the beads by addition of pure acetonitrile. The beads were washed three times with acetonitrile and finally peptides were eluted from the beads with a solution of 2% DMSO in water. Samples were acidified with formic acid to pH4 prior liquid chromatography–mass spectrometry (LC–MS) analysis.

For the in-gel digestion protocol, samples of 50 µg crude venom were first migrated into a SDS polyacrylamide gel. The migration lanes were then sliced in small pieces and washed 10 times in 100 mM ammonium bicarbonate buffer and acetonitrile bathes. Samples were reduced and alkylated with dithiothreitol (50 mM) and iodoacetamide (150 mM), respectively. Proteolysis was performed by incubating the gel pieces in a trypsin/lysC mixture (400 ng, considering a 1:50 enzyme to sample ratio) (Promega) overnight at 37 °C. Digested peptides were finally extracted by successive incubations in 25 mM ammonium bicarbonate, 25 mM ammonium bicarbonate/acetonitrile (1/1, v/v), 5% formic acid and 5% formic acid/acetonitrile (1/1, v/v) buffers. Samples were finally vacuum dried and suspended in trifluoroacetic acid 0.05%, acetonitrile 1% (v/v) in 20 µL water.

### 4.2. Mass Spectrometric Analysis

The LC–MS setup consisted in a Dionex U3000 RSLC liquid chromatography system operated in column switching mode coupled with a Q-Exactive Plus or a Q-Exactive HF mass spectrometer (Thermo Fisher Scientific, Waltham, MA, USA). The samples (200 ng peptide content) were loaded onto a trap column (75 µm × 20 mm, acclaim C18 pepmap 100, 3 µm; Thermo Fisher Scientific, Waltham, MA, USA) by a loading phase (1% acetonitrile, 0.05% trifluoroacetic acid in water) at a flow rate of 5 µL/min. The samples were then eluted onto an analytical column (75 µm × 150 mm, acclaim C18 pepmap 100, 2 µm; Thermo Fisher Scientific, Waltham, MA, USA) by a linear gradient starting from 2% A to 35% B in 66 min. The solvents A and B consisted of water with 0.1% formic acid and acetonitrile with 0.1% formic acid, respectively. The MS acquisition was performed in data dependent acquisition mode. The acquisition loop consisted in a survey scan acquired at a resolution of 70,000 at 200 m/z (60,000 at 200 m/z for the Q-Exactive HF) followed by the selection and the fragmentation of the 12 most intense precursor ions. The resolution of the MS/MS scans was set to 17,500 at 200 m/z (15,000 at 200 m/z for the Q-Exactive HF) and the already fragmented precursor ions were excluded for 30s.

### 4.3. Data Processing

MS data were processed by Peaks studio v.10.0 and v10.5 (Bioinformatics Solutions Inc., Waterloo, Canada) with an NCBI database containing protein sequences known or predicted for *Polistes dominula*, *Vespula germanica* and *Vespula vulgaris* (21.442 entries). In contrast to *Polistes dominula,* no genomic sequences for *Vespula* spp. are known so far. Therefore, *Vespula* spp. samples were also processed with a protein sequence database containing all entries from Hymenoptera order in Uniprot (601.844 entries). Protease specificity was set to trypsin, the error tolerance on the precursor and fragment ions masses were set to 10 ppm and to 0.015 Da, respectively. Carbamidomethylation of cysteine was specified as a fixed modification and oxidation of methionine and acetylation of the N-terminus of the proteins as variable modifications. Identifications were filtered with a false discovery rate of 1% (peptides and proteins) using a database containing reversed sequences.

Resulting proteomic data sets were scanned for double entries and reduced to one entry per protein. GenInfo Identifier (gi) were added where possible. Scanning included a protein BLAST analysis [63] to determine whether duplicate entries are an artifact of using different databases for peptide identification in MS or whether they are truly different proteins. Default search parameters were used (Expect threshold—10; Word size—3; Matrix—BLOSUM62; Gap Costs—Existence: 11 Extension: 1; Compositional adjustments—Conditional compositional score matrix adjustment). GO terms for the biological process, the molecular function as well as the cellular component were assigned to each protein using InterProScan Version 5.39-77.0 [64]. Signal peptides were predicted using SignalP 5.0 with default parameters [39].

### 4.4. Data Availability

All data generated or analysed during this study are included in this article and its Supplementary Material files.

## Figures and Tables

**Table 1:** Identified proteins in *Polistes dominula* and *Vespula* spp. venom that are known allergens.

Identifier	Description	Allergen
AAT95010.1	Allergen Pol d 5 precursor	Pol d 5
NP_001310265.1	Antigen 5 precursor	Pol d 5
XP_015174448.1	Dipeptidyl peptidase IV	Pol d 3/Ves v 3
ACA00159.1	Dipeptidylpeptidase IV preproprotein	Pol d 3/Ves v 3
XP_015179722.1	Hyaluronidase	Pol d 2
P49370	Hyaluronidase A	Ves v 2.0101
Q5D7H4.1	Hyaluronidase B	Ves v 2.0201
AAS67042.1	Phospholipase A1 2 precursor, partial	Pol d 1
AAS67043.1	Phospholipase A1 3 precursor, partial	Pol d 1
Q6Q249.1	Phospholipase A1 4	Pol d 1
AAS67044.1	Phospholipase A1 4 precursor, partial	Pol d 1
Q3ZU95	Phospholipase A1	Ves g 1
P49369	Phospholipase A1	Ves v 1
P35784	Venom allergen 5	Ves g 5
Q05110	Venom allergen 5	Ves v 5
NP_001310266.1	Venom serine protease precursor	Pol d 4

**Table 2:** Identified proteins in *Polistes dominula* and *Vespula* spp. venom that exhibit a signal peptide for transport into the extracellular matrix or have a known function in the venom (“true venom components”). An X indicates in which species the protein was identified. The species for which the protein was originally annotated in the Hymenoptera database is given in square brackets. All *Polistes dominula* proteins were identified by annotated *Polistes dominula* proteins (identified or predicted).

Identifier	Description	PDV	YJV
XP_015182550.1	60S acidic ribosomal protein P2	X	
XP_015183814.1	Acidic phospholipase A2 PA4-like isoform X1	X	
XP_015183815.1	Acidic phospholipase A2 PA4-like isoform X2 [*P. dominula*]	X	X
XP_015181576.1	Alpha-N-acetylgalactosaminidase isoform X1 [*P. dominula*]		X
XP_015181584.1	Alpha-N-acetylgalactosaminidase isoform X2 [*P. dominula*]		X
XP_015184655.1	Apolipophorins [*P. dominula*]	X	X
XP_015183810.1	Apolipoprotein D-like [*P. dominula*]		X
XP_015179728.1	Cyclin-dependent kinase 8-like [*P. dominula*]		X
XP_015182852.1	Digestive cysteine proteinase 1 [*P. dominula*]		X
P0C1M6.1	Dominulin-A	X	
P0C1M7.1	Dominulin-B	X	
XP_015185362.1	FK506-binding protein 2 isoform X1 [*P. dominula*]		X
XP_015185363.1	FK506-binding protein 2 isoform X2 [*P. dominula*]		X
XP_015183963.1	Furin-like protease 1 isoform 1-CRR isoform X1 [*P. dominula*]		X
XP_015183982.1	Furin-like protease 1 isoform 1-CRR isoform X3 [*P. dominula*]		X
XP_015179268.1	Heat shock 70 kDa protein cognate 3 isoform X1 [*P. dominula*]	X	X
XP_015185877.1	Icarapin-like	X	
XP_015186100.1	Interferon-related developmental regulator 1-like [*P. dominula*]		X
XP_015187740.1	Juvenile hormone epoxide hydrolase 1-like	X	
XP_015183311.1	Maltase 1-like [*P. dominula*]		X
P01514	Mastoparan-L [*Vespula lewisii*] *		X
P0C1Q8	Mastoparan-V1 [*V. vulgaris*] *		X
A0A310SDE3	Neuroblastoma suppressor of tumorigenicity 1 [*Eufriesea mexicana*]		X
XP_015173581.1	Nucleobindin-2-like isoform X1 [*P. dominula*]		X
XP_015173583.1	Nucleobindin-2-like isoform X2 [*P. dominula*]		X
XP_015173585.1	Nucleobindin-2-like isoform X3 [*P. dominula*]		X
XP_015173600.1	Peptidylglycine alpha-hydroxylating monooxygenase [*P. dominula*] *		X
XP_015187119.1	Phospholipase A1-like [*P. dominula*]	X	X
XP_015190124.1	Phospholipid hydroperoxide glutathione peroxidase isoform X2 [*P. dominula*]		X
XP_015186079.1	Prosaposin isoform X1 [*P. dominula*]		X
XP_015186089.1	Prosaposin isoform X2 [*P. dominula*]		X
XP_015191732.1	Protein 5NUC-like [*P. dominula*]		X
XP_015181251.1	Protein D2-like [*P. dominula*]		X
XP_015189991.1	Protein disulfide-isomerase A3 [*P. dominula*]		X
XP_015190044.1	Protein lethal(2)essential for life-like	X	
XP_015171801.1	Renin receptor isoform X1 [*P. dominula*]		X
XP_015171802.1	Renin receptor isoform X2 [*P. dominula*]		X
XP_015171803.1	Renin receptor isoform X3 [*P. dominula*]		X
XP_015171804.1	Renin receptor isoform X4 [*P. dominula*]		X
AAP37412.1	Serine protease precursor	X	
A0A1B1JID1	Superoxide dismutase [Cu-Zn] [*A. mellifera*] *		X
A0A0M9A935	Transferrin [*Melipona quadrifasciata*] *		X
XP_015185020.1	Tyramine beta-hydroxylase [*P. dominula*]		X
XP_015179456.1	Uncharacterized protein LOC107067990	X	
XP_015181315.1	Uncharacterized protein LOC107068952	X	
XP_015183315.1	Uncharacterized protein LOC107070028 [*P. dominula*]		X
XP_015185303.1	Vascular endothelial growth factor C	X	
D0EY66	Venom CUB-protease [*V. vulgaris*]		X
A0A3S6I504	Vespakinin [*Parapolybia varia*] *		X
P57672	Vespulakinin-1 [*Vespula maculifrons*] *		X

* Proteins identified based on only one peptide. PDV, *Polistes dominula* venom; YJV, yellow jacket venom. These proteins have an increased likelihood to be false positives.

## Supplementary Materials

The following are available online at https://www.mdpi.com/2072-6651/12/5/323/s1: Table S1: Proteins identified in *Vespula* spp. venom that are venom trace molecules without predicted signal peptide for transport into the extracellular matrix, Table S2: Proteins identified in *Polistes dominula* venom that are venom trace molecules without predicted signal peptide for transport into the extracellular matrix, Table S3: All identified proteins in *Polistes dominula* and *Vespula* spp.
