# Supplementary material for: Shedding Light on the Venom Proteomes of the Allergy-Relevant Hymenoptera Polistes dominula (European Paper Wasp) and Vespula spp. (Yellow Jacket)
Source: Toxins (Basel). 2020 May 14;12(5):323. doi: 10.3390/toxins12050323 (PMC7291082; doi:10.3390/toxins12050323)
Supplement: Supplementary file 1 [file toxins-12-00323-s001.zip › toxins-790682-supplementary proof 1/toxins-790682-supplementary proof 1- Table S1 and Table S2.docx]

Supplementary Materials: Shedding Light on the Venom Proteomes of the Allergy-Relevant Hymenoptera Polistes Dominula (European Paper Wasp) and Vespula spp. (Yellow Jacket)

Johannes Grosch, Christiane Hilger, Maria Beatrice Bilò, Stephanie Kler, Maximilian Schiener,
Gunnar Dittmar, François Bernardin, Antoine Lesur, Markus Ollert, Carsten B. Schmidt-Weber and Simon Blank

**Table S1.** Proteins identified in *Vespula spp*. venom that are venom trace molecules without predicted signal peptide for transport into the extracellular matrix.

| **Identifier** | **Description** | |
| --- | --- | --- |
| XP_015186553.1 | 14-3-3 protein zeta isoform X1 [P. dominula] |  |
| XP_015186554.1 | 14-3-3 protein zeta isoform X2 [P. dominula] |  |
| XP_015182964.1 | 15 kDa selenoprotein [P. dominula] |  |
| XP_015187088.1 | 3-hydroxyacyl-CoA dehydrogenase type-2-like [P. dominula] * |  |
| XP_015179941.1 | 4-coumarate--CoA ligase 1 [P. dominula] |  |
| XP_015174949.1 | 60 kDa heat shock protein mitochondrial-like [P. dominula] |  |
| XP_015182473.1 | Actin-5C [P. dominula] |  |
| XP_015182032.1 | ADP ATP carrier protein [P. dominula] * | |
| XP_015181126.1 | ADP ATP carrier protein-like [P. dominula] | |
| A0A154PH71 | Alcohol dehydrogenase [NADP(+)] A [Dufourea novaeangliae] | |
| XP_015179311.1 | Aldose reductase-like [P. dominula] | |
| XP_015179473.1 | Aldose reductase-like [P. dominula] | |
| A0A088A332 | Alpha-galactosidase [A. mellifera] * | |
| XP_015187594.1 | Annexin B9-like [P. dominula] | |
| XP_015186858.1 | Annexin B9-like isoform X1 [P. dominula] | |
| XP_015186854.1 | Annexin B9-like isoform X2 [P. dominula] | |
| XP_015186855.1 | Annexin B9-like isoform X3 [P. dominula] | |
| T1P687 | Arginine kinase (Fragment) [Trachypus boharti] * | |
| XP_015179151.1 | Aromatic-L-amino-acid decarboxylase isoform X1 [P. dominula] | |
| XP_015179152.1 | Aromatic-L-amino-acid decarboxylase isoform X2 [P. dominula] | |
| XP_015177560.1 | ATP synthase subunit alpha mitochondrial [P. dominula] * | |
| XP_015176975.1 | ATP synthase subunit beta mitochondrial [P. dominula] | |
| XP_015188589.1 | ATP-citrate synthase [P. dominula] | |
| A0A232F3H2 | Carrier domain-containing protein [Trichomalopsis sarcophagae] | |
| A0A0M9A9Y1 | DDB1-and CUL4-associated factor 15 [Melipona quadrifasciata] * | |
| A0A232ETH5 | Delta-aminolevulinic acid dehydratase [Trichomalopsis sarcophagae] | |
| A0A151J5S6 | DNA polymerase (Fragment) [Trachymyrmex cornetzi] | |
| A0A088FHS6 | DNA topoisomerase 1 (Fragment) [Pogonomyrmex vermiculatus] * | |
| A0A0T5 | Dopa decarboxylase (Fragment) [Cleonymus sp. CD007] * | |
| I3QD84 | Dopa decarboxylase [Polyrhachis vicina] * | |
| XP_015184355.1 | E3 SUMO-protein ligase RanBP2 [P. dominula] | |
| XP_015177848.1 | Elongation factor 1-alpha [P. dominula] | |
| XP_015172041.1 | Elongation factor 1-alpha [P. dominula] | |
| XP_015182320.1 | Elongation factor 1-alpha-like [P. dominula] | |
| E2B9R0 | Enolase [Harpegnatos saltator] * | |
| XP_015182123.1 | Farnesol dehydrogenase-like isoform X1 [P. dominula] | |
| XP_015182129.1 | Farnesol dehydrogenase-like isoform X2 [P. dominula] | |
| XP_015188023.1 | Fatty acid synthase [P. dominula] | |
| XP_015184463.1 | Fructose-bisphosphate aldolase-like [P. dominula] | |
| XP_015176829.1 | Fumarate hydratase mitochondrial-like isoform X1 [P. dominula] | |
| XP_015176830.1 | Fumarate hydratase mitochondrial-like isoform X2 [P. dominula] | |
| XP_015186339.1 | Fumarylacetoacetate hydrolase domain-containing protein 2 isoform X1 [P. dominula] | |
| XP_015186347.1 | Fumarylacetoacetate hydrolase domain-containing protein 2A isoform X2 [P. dominula] | |
| XP_015183974.1 | Furin-like protease 1 isoform 1-CRR isoform X2 [P. dominula] | |
| XP_015171805.1 | Glycerol-3-phosphate dehydrogenase [NAD(+)] cytoplasmic-like isoform X1 [P. dominula] | |
| XP_015171806.1 | Glycerol-3-phosphate dehydrogenase [NAD(+)] cytoplasmic-like isoform X2 [P. dominula] | |
| XP_015179269.1 | Heat shock 70 kDa protein cognate 3 isoform X2 [P. dominula] | |
| XP_015177712.1 | Heat shock 70 kDa protein cognate 4 [P. dominula] | |
| XP_015176917.1 | Heat shock 70 kDa protein cognate 5 [P. dominula] | |
| XP_015184783.1 | Heat shock protein 83 [P. dominula] | |
| XP_015178985.1 | Heat shock protein 83-like [P. dominula] | |
| AJG01688.1 | Histone 3 partial [P. dominula] | |
| XP_015173497.1 | Histone H2A [P. dominula] | |
| XP_015179778.1 | Histone H2A [P. dominula] | |
| XP_015182326.1 | Histone H2A.V [P. dominula] | |
| XP_015174052.1 | Histone H2A-like [P. dominula] | |
| XP_015181269.1 | Histone H2B [P. dominula] * | |
| XP_015177524.1 | Histone H2B-like [P. dominula] | |
| XP_015173448.1 | Histone H2B-like [P. dominula] | |
| XP_015174269.1 | Histone H3 [P. dominula] | |
| XP_015172185.1 | Histone H3.3 [P. dominula] | |
| XP_015190876.1 | Histone H4 [P. dominula] | |
| XP_015177662.1 | Histone H4-like [P. dominula] | |
| A0A4S2KL95 | Hypothetical protein DBV15_06798 [Temnothorax longispinosus] | |
| A0A4S2KCD8 | Hypothetical protein DBV15_06872 [Temnothorax longispinosus] | |
| A0A195BL10 | Insulin-like growth factor-binding protein complex acid labile subunit [Atta colombica] | |
| XP_015178622.1 | Leucine-rich PPR motif-containing protein mitochondrial-like isoform X1 [P. dominula] | |
| XP_015178623.1 | Leucine-rich PPR motif-containing protein mitochondrial-like isoform X2 [P. dominula] | |
| A0A0C9RIC3 | NAGK protein [Fopius arisanus] * | |
| XP_015188288.1 | Peroxiredoxin 1-like [P. dominula] | |
| XP_015188423.1 | Probable ATP-dependent RNA helicase ddx20 [P. dominula] | |
| XP_015184835.1 | Probable enoyl-CoA hydratase mitochondrial [P. dominula] | |
| XP_015190123.1 | Probable phospholipid hydroperoxide glutathione peroxidase isoform X1 [P. dominula] | |
| XP_015185022.1 | Putative leucine-rich repeat-containing protein DDB_G0290503 [P. dominula] | |
| XP_015171575.1 | Pyruvate carboxylase mitochondrial isoform X1 [P. dominula] | |
| XP_015171576.1 | Pyruvate carboxylase mitochondrial isoform X2 [P. dominula] | |
| A0A0C9Q2V5 | SesB_0 protein [Fopius arisanus] * | |
| A0A0C9Q032 | SesB_1 protein [Fopius arisanus] * | |
| XP_015173601.1 | SKI/DACH domain-containing protein 1-like [P. dominula] | |
| XP_015184237.1 | Sodium-dependent neutral amino acid transporter B(0)AT3 isoform X1 [P. dominula] | |
| XP_015184238.1 | Sodium-dependent neutral amino acid transporter B(0)AT3 isoform X2 [P. dominula] | |
| XP_015181039.1 | Stress-induced-phosphoprotein 1 [P. dominula] | |
| K7JU27 | Thioredoxin domain-containing protein [Nasonia vitripennis] * | |
| A0A232FHM8 | Thioredoxin domain-containing proteinn [Trichomalopsis sarcophagae] * | |
| A0A0N0BBR6 | Transcription elongation factor B polypeptide 2 [Melipona quadrifasciata] * | |
| XP_015179360.1 | Tubulin alpha-1 chain [P. dominula] * | |
| XP_015171970.1 | Tubulin beta-1 chain [P. dominula] | |
| E9IEU1 | Uncharacterized protein (Fragment) [Solenopsis invicta] * | |
| A0A195FMR3 | Uncharacterized protein (Fragment) [Trachymyrmex septentrionalis] * | |
| XP_015174135.1 | Uncharacterized protein LOC107065198 [P. dominula] | |
| XP_015176990.1 | Uncharacterized protein LOC107066664 isoform X1 [P. dominula] | |
| XP_015176998.1 | Uncharacterized protein LOC107066664 isoform X2 [P. dominula] | |
| XP_015183284.1 | Uncharacterized protein LOC107070011 [P. dominula] | |
| XP_015190535.1 | Uncharacterized protein LOC107074034 [P. dominula] | |
| A0A088AGJ8 | Uncharacterized protein [A. mellifera] | |
| A0A087ZYM2 | Uncharacterized protein [A. mellifera] * | |
| A0A158NXA1 | Uncharacterized protein [Atta cephalotes] * | |
| A0A151I8A0 | Uncharacterized protein [Cyphomyrmex costatus] * | |
| A0A151IEW8 | Uncharacterized protein [Cyphomyrmex costatus] * | |
| K7J5G8 | Uncharacterized protein [Nasonia vitripennis] | |
| K7JS66 | Uncharacterized protein [Nasonia vitripennis] * | |
| A0A151JQJ4 | Uncharacterized protein [Trachymyrmex cornetzi] * | |
| A0A151IZY3 | Uncharacterized protein [Trachymyrmex cornetzi] * | |
| A0A151IZ73 | Uncharacterized protein [Trachymyrmex cornetzi] * | |
| A0A151ITL6 | Uncharacterized protein [Trachymyrmex cornetzi] * | |
| A0A151JUA0 | Uncharacterized protein [Trachymyrmex septentrionalis] * | |
| A0A195EQ16 | Uncharacterized protein [Trachymyrmex septentrionalis] * | |
| A0A151JWR0 | Uncharacterized protein [Trachymyrmex septentrionalis] * | |
| A0A232EIG7 | Uncharacterized protein [Trichomalopsis sarcophagae] | |
| P57672 | Vespulakinin-1 [Vespula maculifrons] * | |
| A0A158NJ59 | WD_REPEATS_REGION domain-containing protein [Atta cephalotes] * | |

The species for which the protein was originally annotated in the Hymenoptera database is given in square brackets. *Proteins identified based on only one peptide. These proteins have an increased likelihood to be false positives.

**Table S2.** Proteins identified in *Polistes dominula* venom that are venom trace molecules without predicted signal peptide for transport into the extracellular matrix.

| **Identifier** | **Description** |
| --- | --- |
| XP_015172728.1 | 14-3-3 protein epsilon |
| XP_015187088.1 | 3-hydroxyacyl-CoA dehydrogenase type-2-like |
| XP_015174949.1 | 60 kDa heat shock protein, mitochondrial-like |
| XP_015179141.1 | Actin, clone 205-like |
| XP_015179142.1 | Actin, muscle |
| XP_015182473.1 | Actin-5C |
| XP_015179311.1 | Aldose reductase-like |
| XP_015182895.1 | Ankyrin repeat domain-containing protein SOWAHC * |
| XP_015181613.1 | Apoptotic chromatin condensation inducer in the nucleus |
| XP_015188027.1 | Arginine kinase |
| XP_015177560.1 | ATP synthase subunit alpha, mitochondrial |
| XP_015176975.1 | ATP synthase subunit beta, mitochondrial |
| XP_015172129.1 | Bipolar kinesin KRP-130-like |
| XP_015172251.1 | Calponin homology domain-containing protein DDB_G0272472-like isoform X4 * |
| XP_015184337.1 | Cofilin/actin-depolymerizing factor homolog |
| XP_015178906.1 | Delta-1-pyrroline-5-carboxylate dehydrogenase, mitochondrial |
| P0C1M6.1 | Dominulin-A |
| P0C1M7.1 | Dominulin-B |
| XP_015177848.1 | Elongation factor 1-alpha |
| AJG01714.1 | Elongation factor 1-alpha F2, partial |
| ACB70872.1 | Elongation factor 1-alpha, partial |
| XP_015182320.1 | Elongation factor 1-alpha-like |
| XP_015181294.1 | Farnesol dehydrogenase-like |
| XP_015182123.1 | Farnesol dehydrogenase-like isoform X1 |
| XP_015182129.1 | Farnesol dehydrogenase-like isoform X2 |
| XP_015188023.1 | Fatty acid synthase |
| XP_015186347.1 | Fumarylacetoacetate hydrolase domain-containing protein 2A isoform X2 |
| XP_015178478.1 | Glyceraldehyde-3-phosphate dehydrogenase 2 |
| XP_015179269.1 | Heat shock 70 kDa protein cognate 3 isoform X2 |
| XP_015177712.1 | Heat shock 70 kDa protein cognate 4 |
| XP_015174688.1 | Heat shock 70 kDa protein cognate 4-like |
| XP_015184783.1 | Heat shock protein 83 |
| XP_015186657.1 | Heat shock protein beta-1 isoform X2 |
| XP_015186056.1 | Helicase POLQ-like isoform X1 * |
| XP_015186066.1 | Helicase POLQ-like isoform X2 * |
| AJG01688.1 | Histone 3 partial * |
| XP_015177598.1 | Histone H2A * |
| XP_015182326.1 | Histone H2A.V * |
| XP_015174052.1 | Histone H2A-like * |
| XP_015172623.1 | Histone H3 * |
| XP_015172185.1 | Histone H3.3 * |
| XP_015190876.1 | Histone H4 |
| XP_015177662.1 | Histone H4-like |
| AEN62318.1 | IRP30, partial |
| XP_015171673.1 | Isocitrate dehydrogenase [NADP] cytoplasmic |
| XP_015172246.1 | Myb-like protein P isoform X1 * |
| XP_015172247.1 | Myb-like protein P isoform X2 * |
| XP_015172250.1 | Myb-like protein P isoform X3 * |
| XP_015172252.1 | Myb-like protein P isoform X5 * |
| XP_015183299.1 | Na(+)/H(+) exchange regulatory cofactor NHE-RF1 |
| XP_015180982.1 | NADP-dependent malic enzyme isoform X3 |
| XP_015188288.1 | Peroxiredoxin 1-like |
| XP_015187116.1 | Phospholipase A1-like |
| XP_015187123.1 | Probable ATP-dependent RNA helicase DDX55 |
| XP_015184835.1 | Probable enoyl-CoA hydratase mitochondrial |
| XP_015174225.1 | Probable medium-chain specific acyl-CoA dehydrogenase, mitochondrial |
| XP_015179974.1 | Prostaglandin reductase 1-like |
| XP_015173092.1 | Protein lethal(2)essential for life-like |
| XP_015173078.1 | Protein lethal(2)essential for life-like |
| XP_015184444.1 | Protein PFF0380w |
| XP_015171467.1 | Receptor expression-enhancing protein 5-like |
| XP_015180682.1 | Reticulon-1-A isoform X3 |
| XP_015180683.1 | Reticulon-1-A isoform X4 |
| XP_015180684.1 | Reticulon-1-A isoform X5 |
| XP_015180685.1 | Reticulon-1-A isoform X6 |
| XP_015180686.1 | Reticulon-1-A isoform X7 |
| XP_015180674.1 | Reticulon-4 isoform X1 |
| XP_015180675.1 | Reticulon-4 isoform X2 |
| XP_015172957.1 | Serine/arginine-rich splicing factor 1B |
| XP_015185566.1 | Sorting nexin-2 |
| XP_015174910.1 | Toll-like receptor 8 |
| XP_015179091.1 | Tubulin alpha chain-like * |
| XP_015179615.1 | Uncharacterized oxidoreductase YjmC |
| XP_015187606.1 | Uncharacterized protein LOC107072309 |
| XP_015188113.1 | Uncharacterized protein LOC107072577 isoform X1 |
| XP_015188114.1 | Uncharacterized protein LOC107072577 isoform X2 |
| XP_015179524.1 | Voltage-dependent anion-selective channel-like |

*Proteins identified based on only one peptide. These proteins have an increased likelihood to be false positives.
